# PUFA modulation of ASIC3 involves both specific and lipid solvent-like interactions

**DOI:** 10.64898/2026.01.02.697424

**Published:** 2026-01-02

**Authors:** Rebecca Roth, Ramya Bandarupalli, Robert C. Klipp, Jing Li, John R. Bankston

**Affiliations:** 1 Department of Physiology and Biophysics, University of Colorado Anschutz Medical Campus, Aurora, CO, USA.; 2 Department of Biomolecular Sciences, University of Mississippi, Oxford, MS USA.

**Keywords:** Acid-sensing ion channels, Polyunsaturated Fatty Acids, Arachidonyl Glycine, Docosahexaenoic Acid

## Abstract

Inflammatory mediators including polyunsaturated fatty acids (PUFAs) are known to potentiate ASIC3 by inducing changes in multiple gating features, yet the molecular basis for their interactions remains poorly defined. Using all-atom MD simulations and electrophysiology, we show that DHA accumulates around ASIC3 through loosely coordinated interactions with a membrane-facing electropositive region along the outer leaflet of TM1. In the open state, the carboxylate head group strongly binds to a critical arginine (R63) along with nearby polar residues that are necessary to slow the rate in channel desensitization rate but not to increase the pH sensitivity for channel activation. Moreover, mutating R63 disrupted effects on ASIC3 desensitization induced by PUFAs but not N-acyl amino acids (NAAAs) or lysophosphatidylcholines (LPCs). Our results provide the first detailed description of a functional PUFA binding site on ASICs, offering new insights into lipid modulation and potential strategies for developing novel therapeutics for pain and inflammation.

## INTRODUCTION

Cell membranes are composed of a wide diversity of lipids that can have profound impacts on the activity of membrane proteins. There is a long history describing the regulation of ion channel function by the lipid environment but there is little agreement on mechanisms that influence protein-lipid interactions or channel regulation^1–6^. In general, channel regulation is thought to occur through two major mechanisms: indirect effects on conformational rearrangements through changes in the lipid bilayer (i.e. thickness, fluidity, curvature), or alterations to channel gating through stable interactions. Characterizing the interactions between lipids and ion channels has become even more complex^7–9^. Several hypotheses have been presented including stable binding of lipids to a binding site on the channel, non-selective transmembrane domain solvation, and/or the formation of local lipid ring (annulus) around the channel^10–13^. Understanding these dynamic interactions remains a critical challenge in the field, highlighting the need for both experimentation and computational approaches to disentangle their relative contributions influencing channel gating and modulation.

Over the last 20 years, there has been increasing evidence that Acid-Sensing Ion Channels (ASICs) are critically modulated by a number of different lipids^3,14–16^. ASICs are pH-activated, sodium-permeable ion channels that belong to the Deg/ENaC family of ion channels and are highly expressed in the central (CNS) and peripheral (PNS) nervous systems where they canonically serve as pH sensors^17,18^. The ASIC3 isoform is the most sensitive to proton-mediated activation and is predominantly expressed in nociceptive sensory neurons^19,20^. Inhibiting ASIC3 activity through pharmacological blockade or genetic deletion has been shown to prevent the development of chronic muscle and tactile hyperalgesia in response to repeated injections of acid, formalin, reserpine, and carrageenan^21–23^. Proinflammatory mediators released under these conditions including neurotrophins (i.e. NGF), cytokines, serotonin, and lipids, have been shown to increase sensory neuronal excitability through modulation of ASICs^14,22,24^. Work from our lab and others have identified at least three classes of single acyl chain lipids that can potentiate ASIC3 currents: polyunsaturated fatty acids (PUFAs), and lysophosphatidylcholines (LPCs), and N-acyl amino acids (NAAAs)^3,14,15^. While structurally distinct, all three lipid classes exert similar effects on the channel. Each lipid slows the rate of channel desensitization, shifts the pH dependence of activation in the basic direction, and increases the current even at saturating proton concentrations^3,14,15^. A careful examination of the structural requirements for lipid regulation of ASIC3 suggested roles for both the head and tail group of the lipid^3^. However, the electronegativity of the head group demonstrated to be the most critical element in determining the strength of potentiation.

PUFA concentrations increase during many pathological conditions such as ischemia, epilepsy, tissue injury, and inflammation^25–27^. Their release from phospholipids via activation of phospholipases during these events can allow PUFAs to move freely through cells to influence signaling pathways that regulate gene expression, receptor signaling, and membrane dynamics^28^. Additionally, PUFAs can be metabolized into analogs which can either have similar or opposing effects on ion channel regulation in comparison to their precursors, depending on their new structural properties. Marra and colleagues demonstrated that inflammatory exudates with neutral pHs from human patients were also sufficient to activate ASICs including ASIC3^14^. The active components of the exudates were found to be two different classes of single acyl chain lipids that were present at high concentrations: an ω-6 PUFA known as arachidonic acid (AA) and LPC. The combination of these lipids was sufficient to activate ASIC3 in the absence of acidification, and injections of LPC and AA into mouse hind paws produced significant pain behaviors that were reduced in ASIC3 KO mice or mice co-injected with an ASIC3-specific inhibitor APETx2. This provides evidence that ASIC3 may be a viable target for pharmacological inhibition in inflammatory pain, which requires a better understanding of the interactions between these lipids and the channel.

Work from our lab and others has pointed to a region on TM1 near the outer leaflet as potentially being critical for lipid regulation of ASIC^3^. However, these studies provide an incomplete picture. We previously mutated several residues in this region and showed a reduced effect of the PUFA docosahexaenoic acid (DHA) but did not have evidence of other potential interactions. Molecular dynamics (MD) simulations on human ASIC3 suggested a pair of arginine’s near the outer leaflet may be critical, but one of those arginine residues is not present in other isoforms and orthologues that are still sensitive to lipid modulation^29^. To overcome these limitations, we combined Molecular Dynamics and patch clamp electrophysiology to determine the residues on ASIC3 that may be critical for lipid interaction and functional regulation of the channel. We discovered several residues that are required for both interaction and the change in channel desensitization that accompanies lipid modulation. Interestingly, those same mutations do not prevent the lipid-mediated change in channel sensitivity to protons. Overall, we hypothesize that the data we present here points to complex regulation of the channel by lipids and that a model where the lipid stably binds to the channel is likely insufficient to describe modulation of ASIC3 by single acyl chain lipids.

## Results

### DHA potentiates ASIC3 activation through preferential binding to the extracellular leaflet:

**Figure 1.**
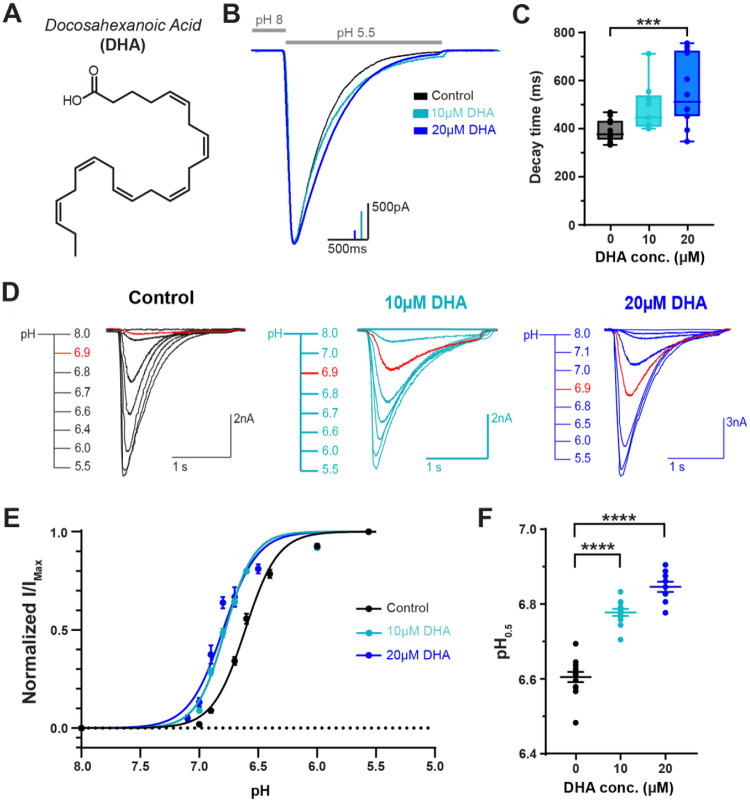
DHA potentiates ASIC3 gating effects in a dose-dependent manner. **(A)** Chemical structure of DHA, a carboxylic acid with a 22-carbon chain and 6 cis double bonds starting from the 3^rd^ carbon. **(B)** Representative whole-cell recordings showing the slowing of the desensitization rate of ASIC3 ± 10 and 20μM DHA. Currents were elicited by performing fast-switch perfusion changes between pH 8 and pH 5.5 solutions. **(C)** Box plot showing the time point at which ASIC3 currents decayed from their peak by 63% (1/e) at 0, 10 and 20μM DHA. **(D)** Representative whole-cell recordings showing pH-dependent activation of ASIC3 ± 10 and 20μM DHA. Traces elicited at pH 6.9 are highlighted in red to visualize the increase in pH sensitivity in the presence of DHA concentrations. **(E)** Fitted curves showing the pH dependence of activation of ASIC3 WT at different concentrations of DHA, fitted using a Hill-type equation (see Equation 1 in Methods). **(F)** Average activation pH_0.5_ values for ASIC3 channels in response to 0, 10, and 20μM DHA. All data given as mean ± SEM. C-D, One-way ANOVA post hoc Dunnett’s test. ***, P < 0.001; ****, P < 0.0001 (see Methods and Table 1 for details).

DHA, the most abundant PUFA in the brain, acts as a strong potentiator of both ASIC1 and ASIC3 channels^3,15^. Structurally, DHA contains a carboxyl head group and a 22-carbon chain tail group which contains 6 cis double bonds throughout the acyl chain (Fig. 1A). Previous work on PUFA modulation of ASICs from our group and others has largely focused on the other abundant PUFA in the brain: arachidonic acid (AA). However, DHA exhibits much stronger potentiating effects on ASIC3 gating including a larger shift in the pH-dependence of activation and a larger slowing of channel desensitization. All PUFAs have the same carboxyl head group which we have shown is the primary driver of the potentiation, so we elected to focus this study on DHA as a model lipid for understanding, generally, how PUFAs bind to and modulate ASIC3 channels.

To confirm and extend on our previous measurements of the modulatory effects of DHA on ASIC3 channels, we performed whole-cell patch-clamp recordings in Chinese hamster ovary (CHO) cells transfected with rat ASIC3 (rASIC3) and measured currents in the absence and presence of 10 or 20μM DHA. To ensure the effect was saturated, cells were preincubated for at least 5 minutes in DHA-containing pH 8 solution prior to each recording. We previously showed that the effects of DHA were largely saturated in 1–2 minutes^3^. Consistent with our previous work, the application of DHA leads to multiple changes in channel function. First, addition of both 10 and 20μM DHA resulted in a consistent and reproducible slowing of channel desensitization rate which can be seen when we overlay currents elicited by a fast solution switch from pH 8 to pH 5.5 (Fig. 1B). To quantify the slowing of channel desensitization, we measured the time it takes for the current to decay to 1/e (63%) of the peak value. Application of 10 and 20μM slowed the rate of channel desensitization in a concentration-dependent manner, slowing the rate from 390 ms to 485 and 558 ms respectively (Fig. 1C, Supplementary Table 1). In addition to a change in desensitization rate, 10 and 20μM concentrations of DHA also increased the proton sensitivity of channel activation. To demonstrate this, we made solution switches from pH 8 to varying pH levels between 7.0 and 5.5 (Fig. 1D) and then plotted the peak current as a function of the activating pH (Fig. 1E). Consistent with our previous findings, ASIC3 currents show a half-maximally activating pH value (pH_0.5_) of 6.6 in the absence of lipid. In the presence of 10μM and 20μM DHA, the pH_0.5_ exhibited an alkaline shift by about 0.17 and 0.24 pH units respectively (Fig. 1F, Supplementary Table 1).

It has been hypothesized for many ion channels, including ASICs, that the negatively charged head group of PUFAs likely makes stable electrostatic interactions or hydrogen bonds with side chain atoms of arginine (R), lysine (K), and tyrosine (Y) residues located along their TM segments to alter channel gating^6,30,31^. Both TM1 and TM2 of ASIC3 have positively charged residues positioned near the inner and outer leaflet of the channel, indicating the possibility that there are interaction sites for lipids with negatively charged head groups on either or both sides of the channel. It has been shown that AA shifts the pH-dependence of ASIC2a channel activation upon application to either the intracellular or extracellular side of the channel, while we previously showed that a PUFA derivative of AA known as N-arachidonyl glycine (AG) only produces potentiating effects on ASIC3 when applied to the outer leaflet of the membrane^3,15^. Therefore, we asked whether PUFAs exhibited a sidedness for potentiating ASIC3 channels.

**Figure 2:**
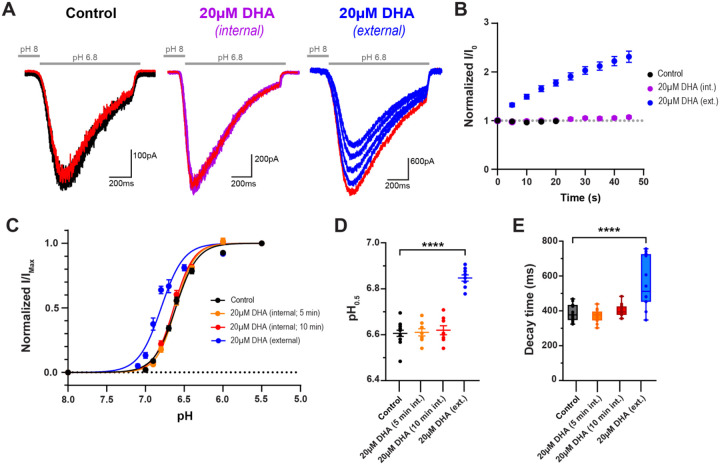
Exposure to the extracellular leaflet is necessary to induce DHA-mediated potentiation of ASIC3 currents. **(A)** Representative traces of the first 5 sweeps within a single cell showing repeated pH 6.8-evoked currents in the absence (right) or presence of either internal (middle) or external (left) application of 20μM DHA. The red trace represents the current elicited during the 5^th^ sweep of the time course in B. **(B)** Time course of pH 6.8-evoked ASIC3 currents. Internal application represents 20μM DHA within the patch pipette; external applications represent 20μM DHA within the pH 8 bath solution (*n* = 7–10 cells/ condition). **(C)** Fitted curves showing the pH dependence of activation of ASIC3 WT following different applications of 20μM DHA, fitted using a Hill-type equation (see Equation 1 in Methods). **(D)** Average activation pH_0.5_ values for ASIC3 channels in response to internal or external application of 20μM DHA. **(E)** Box plot showing the time point at which ASIC3 currents decayed from their peak by 63% (1/e) in response to internal or external application of 20μM DHA.C-E, ASIC3 WT and external 20μM DHA are replotted from Fig. 1 for comparison. All data given as mean ± SEM. D-E, One-way ANOVA post hoc Dunnett’s test. ****, P < 0.0001 (see Methods and Table 2 for details).

Here, we tested the hypothesis that DHA potentiates ASIC3 currents only when it is applied to the extracellular side of the channel. To do this, we applied 20μM DHA to the patch pipette and elicited channel activation by applying brief external applications of a mildly activating pH 6.8 solution (Fig. 2A). We saw no changes in the peak current across several sweeps when compared to control conditions, whereas acute external application of 20μM DHA produced a 2.3-fold increase in current amplitude size within 45 seconds of exposure (Fig. 2B). It is possible that the time it takes to start the recording resulted in saturation of the effect prior to our measurement, thus, we also looked at changes in the steady state parameters. There were no significant changes to the pH sensitivity for channel activation or the rate in channel desensitization when DHA was internally applied for 5 or 10 minutes (Fig 2C–E, Supplementary Table 2). Altogether, this strongly suggests that DHA, like AG, alters ASIC3 channel desensitization and pH sensitivity through action on the extracellular side of the channel.

### DHA accumulates near ASIC3, but channel opening exposes an arginine for stable binding:

Work from our lab and others have suggested that a cluster of arginine residues located in the outer leaflet of TM1 are potentially critical for mediating lipid binding to ASIC1 and ASIC3^3,29^. However, previous MD simulations and electrophysiology data support differing conclusions about which outer leaflet residues are critical for functional regulation of the channel. Therefore, to better understand how lipid interactions with ASICs lead to changes in function, we employed all-atom MD simulations to determine, at atomic resolution, how DHA interacts with the TMs of ASIC3 in different conformational states. With the predictions from these models, we then performed site-directed mutagenesis and patch-clamp electrophysiology to understand the impact these residues have on lipid regulation of the channel.

**Figure 3.**
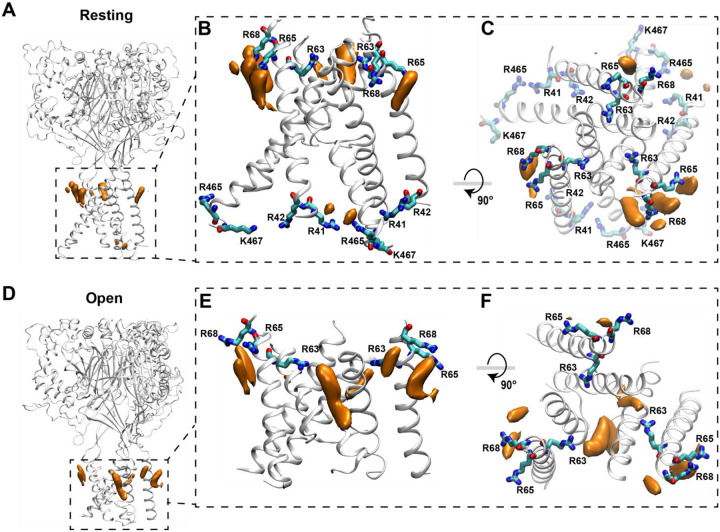
Spontaneous binding of DHA is more stable when ASIC3 is in an open state. **(A)** Side view of the full ASIC3 resting state homology model. **(B)** Side view and **(C)** top view of the resting state hASIC3 model showing spontaneous accumulation of DHA around both the intracellular and the extracellular ends of the TM helices. DHA preferentially localizes near the arginine-rich region formed by residues R65 and R68 (cyan), creating a lipid density in the outer leaflet of the membrane. Additional DHA densities are seen near residues R41, R42, R465, and K467, extending toward the intracellular leaflet. **(D)** Side view of the full ASIC3 open state homology model. **(E)** Side view and **(F)** top view of the open state hASIC3 model showing spontaneous accumulation of DHA around only the extracellular ends of the TM helices. Densities were calculated using VMD and visualized as isosurfaces at an isovalue of 0.4.

To perform MD simulations, homology models of human ASIC3 (hASIC3) were generated based on the chicken ASIC1 structure in the open state (PDB ID 4NTY) and the human ASIC1 structure in the resting state (PDB ID 7CFS) as no structures of ASIC3 have been solved to date^32,33^. To probe spontaneous lipid-protein interactions, DHA molecules were randomly distributed within the lipid bilayer at a 10:1 POPC: DHA molar ratio (Fig. 3, Table 1; see Methods). The systems were equilibrated and subsequently simulated for multi-microsecond timescales to identify DHA-interaction hotspots on hASIC3. During these simulations, DHA molecules diffused within the lipid bilayer and began to accumulate at sites on the channel near both the inner and outer leaflet of the membrane (Fig. 3A,D). In the rest state, densities representing the head group of DHA can been seem accumulating at sites both on the intracellular and extracellular side of the channel (Fig. 3B,C). DHA clustering was most pronounced around a pair of adjacent positively charged residues R65 and R68 located in the outer leaflet of TM1, which appear to form a distinct electropositive region that attracted the negatively charged DHA headgroups. Additionally, the head group of DHA molecules also clustered near positively charged residues in the intracellular leaflet including R41, R42, R465, and K467. Notably, DHA interactions with inner-leaflet residues were observed only in the resting-state trajectory, as the intracellular arginine residues were unresolved in the 4NTY structure used for homology modeling of the open state.

In the open state, a large density absent in the resting state appeared near residue R63 (Fig. 3E, F). DHA molecules continued to interact with R65 and R68, but accumulated much more densely around R63, forming a distinct higher-density lipid cluster at the outer membrane interface. This difference is likely due to its accessibility; in the resting state, R63 is positioned deep within the protein interior, shielded from the surrounding lipid bilayer. When the channel opens, TM1 moves outward, repositioning R63 toward the membrane interface and exposing its positively charged side chain to the lipid environment. This structural rearrangement creates a new electropositive binding surface that allows the DHA headgroup to engage with R63 directly through stable electrostatic interactions.

**Figure 4.**
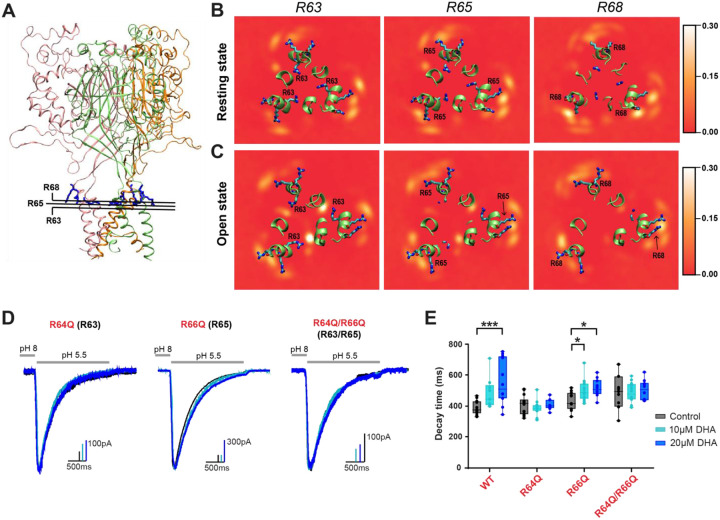
Mutation of R63 eliminates the effect of DHA on channel desensitization. **(A)** Cross-sectional view of the ASIC3 trimer highlighting the levels used to generate the occupancy maps in panels B and C. The protein is shown in ribbons with three subunits colored pink, orange, and green. 2D volume maps illustrate the DHA occupancy near residues R63, R65, and R68 in the resting **(B)** and open **(C)** state simulations. The color scale represents the fractional occupancy of DHA, where a value of 0.3 corresponds to ⩾ 30% occupancy during the simulation. Lighter to white regions indicate higher DHA density. **(D)** Representative whole-cell recordings showing the slowing of the desensitization rate of ASIC3 mutants ± 10 and 20μM DHA. **(E)** Box plot showing the time point at which ASIC3 mutant currents decayed from their peak by 63% (1/e) at 0, 10 and 20μM DHA. ASIC3 WT data are replotted from Fig. 1 for comparison. All data given as mean ± SEM. C-D, One-way ANOVA post hoc Dunnett’s test. *, P < 0.05; ***, P < 0.001 (see Methods and Table 4 for details).

To look more quantitatively at the interaction interface for DHA on the extracellular side of the channel, we calculated time-averaged occupancy maps which reflect the fraction of time that the head group of DHA molecules occupied a particular position in space relative to each individual residue (Fig. 4A–C). These heat maps show an increased concentration of DHA molecules surrounding the channel centered at these arginine residues. In addition, using a distance vs. time plot showing the proximity of the carboxylate carbon of DHA and the terminal carbon to the side chain (Supplementary Figures 1–3), we calculated the DHA contact duration at residues R63, R65, and R68 in both states. In both the open and resting state simulations, DHA molecules were recruited to this region throughout the 5-μs simulations, yet their distributions differed markedly between two states. In the resting state, DHA primarily interacted with R65 and R68 (Fig. 4B–C), exhibiting mean lifetimes of 162 and 124 ns, respectively, and the longest individual binding events lasting up to 1.2 and 2.9 μs (Table 2). DHA density near R63 was minimal in the resting conformation and no detectable binding events were observed during the 5-μs trajectory, reflecting its buried position (Fig. 4B, Table 2).

In contrast, in the open state, R63 became exposed to the membrane and formed stable interactions with DHA, characterized by fewer total events but much longer durations. The mean duration of these binding events were about 495 ns, with the longest individual binding event lasting over 3 μs (Table 2). Meanwhile, R65 and R68 continued to form dynamic, recurrent interactions, with mean lifetimes of 142 and 113 ns, respectively, but were more diffuse and less persistent (Supplementary Figure 3, Table 2). This is also reflected within the occupancy maps which show less DHA density hot spots near R65 and R68 in the open state when compared to R63 (Fig. 4C). Interestingly, the occupancy at R63 in the open state exceeded that observed at R65 and R68 in either conformational state (Table 2), suggesting that this residue represents the primary binding hotspot within the arginine cluster located on the outer leaflet.

### Mutation of a single arginine prevents DHA-mediated slowing in desensitization rate:

To validate the predictions of our simulated models, we created mutations to residues identified as hotspots and measured their impacts on DHA-mediated slowing of channel desensitization. Given that DHA acts on ASIC3 currents only when applied to the extracellular side of the channel, we focused our mutagenesis on the arginine residues located near the outer leaflet of the membrane. Mutations were made using the rat ASIC3 gene to stay consistent with our previous work and to ensure optimal current magnitudes within our cell system because, in our hands, rat ASIC3 generates larger currents when compared to human ASIC3 and many of the mutations made along the TMDs tend to greatly reduce expression (Table 3). Because the residue numbers differ slightly in rat versus human ASIC3 (Supplementary Figure 4), we have distinguished residue numbers within each figure by color with human residues displayed in black and rat residues in red.

The first arginine found in our simulation, R68, is only present in the human orthologue. This arginine is not present in any of the ASIC isoforms or orthologues that have been confirmed to be regulated by single acyl chain lipid including human ASIC1a, rat ASIC1a, rat ASIC2a, and rat ASIC3 suggesting that it is not necessary for lipid potentiation of the channel^3,14,15^. To examine the other two arginine residues in rASIC3, R64 and R66 (R63 and R65 in hASIC3), we made 3 mutant channels that would neutralize these sites: two of which introduced single-point mutations at each site (R64Q and R66Q), and one of which introduced mutations to both sites (R64Q/R66Q). All mutations produced functional channels that showed proton activated currents (Fig. 4D), although the channels that contain the R64Q mutation showed smaller current amplitudes. To assess the changes in channel desensitization rate, we measured the time it took for the current to decay from the peak of activation to 1/e for each mutant channel in the absence and presence of 10 or 20μM DHA (Fig. 4E, Supplementary Table 3). When comparing the effects of DHA on each mutant channel, we found that the average decay rates for R64Q/R66Q double mutant channels did not change following the application of DHA at either concentration. When assessing the single-point mutant channels, R64Q was sufficient to eliminate the effect of DHA on channel desensitization at both concentrations while R66Q was not. This suggests that R64, but not R66, is required to mediate the effects of DHA on ASIC3 channel desensitization.

In combination with our MD simulations, these findings indicate that the exposure of R63 in human ASIC3 upon channel opening creates a more stable interaction site for DHA, while R65 and R68 form an adjacent electropositive surface that may facilitate DHA recruitment. DHA binding near R63 at the inter-subunit interface in the open state potentially helps stabilize the open conformation of the ASIC channel. This state-dependent binding of DHA, favoring the open over the resting conformation, offers a structural explanation for its experimentally observed potentiation on ASIC3 desensitization kinetics.

### Molecular docking reveals other key residues which mediate effects on desensitization:

The spontaneous binding simulations showed that DHA molecules accumulate near the extracellular end of TM1, forming long-lived interactions with a cluster of arginine residues, particularly near R63 in the open state. To further assess whether nearby residues contribute to DHA stabilization and to characterize the persistence of headgroup interactions, we performed molecular docking followed by all-atom MD simulations. In contrast to the random DHA partitioning approach, which allowed DHA to freely explore the membrane environment, molecular docking enables targeted placement of DHA at the three equivalent binding sites centered on R63 and thereby allowing a systematic evaluation of DHA interactions with surrounding residues during extended MD simulations.

**Figure 5.**
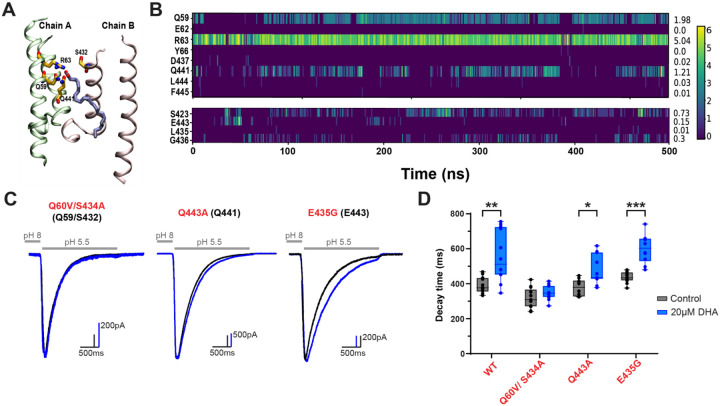
Other key residues nearby R63 contribute to PUFA interaction with ASIC3. **(A)** Molecular visualization from trajectory 3 showing DHA bound near the TM region. The protein is shown in ribbons with two chains colored lime and pink. DHA is displayed in ice-blue licorice, and the residues from both chains that interact with DHA are shown in orange licorice representation. **(B)** Heatmap showing the DHA-residue contact analysis throughout the simulation. The X-axis represents simulation time, while the Y-axis (left) lists the residues that formed contacts with DHA. The color intensity reflects the contact number for each frame. The right side of the Y-axis indicates the average contact number, defined as the mean number of atomic pairs within 4 Å between DHA and each residue across the entire trajectory 3. **(C)** Representative whole-cell recordings showing the slowing of the desensitization rate of ASIC3 mutants ± 20μM DHA. **(D)** Box plot showing the time point at which ASIC3 mutant currents decayed from their peak by 63% (1/e) at 0 and 20μM DHA. ASIC3 WT data are replotted from Fig. 1 for comparison. All data given as mean ± SEM. D, unpaired t-test with Welch’s correction. *, P < 0.05; **, P < 0.01; ***, P < 0.001 (see Methods and Table 5 for details).

Contact analysis of the docked MD trajectories revealed that, in addition to the persistent interaction with R63, the DHA headgroup frequently engaged in contacts with several nearby polar residues (Fig. 5A–B). The contact number, defined as the number of atomic pairs between DHA and a residue that are within 4 Å in each simulation frame, was used to quantify the strength and persistence of these interactions over time. Notably, Q59 and Q441 displayed average contact numbers greater than 1, indicating that multiple atomic pairs between these residues and the DHA carboxylate group remain within proximity. S432 also interacted frequently with DHA, showing the average contact number as 0.73, which is significantly higher than other nearby residues such as E433 and D437. Additionally, multiple detectable binding events were made at Q59 and S432 with randomly partitioned DHA molecules throughout both open and resting state simulations that produced Figures 3 and 4, while binding events were only detectable at Q441 in the open state (Table 2).

To assess their functional involvement in DHA-mediated potentiation of ASIC3, we again made point mutations to these equivalent residues in rat ASIC3 and measured the desensitization rates from currents elicited at pH 5.5 in the absence and presence of 20μM DHA. As a negative control for the model, we also chose to mutate a residue located near R64 in rat ASIC3 but showed little to no interaction with the head group of DHA throughout the simulations (E435). In agreement with the model, mutations to Q60 and S434 together in rASIC3 significantly reduced the effect of DHA on the desensitization rate of the channel, only producing a slight change in decay rate by 30ms (Fig. 5C–D, Supplementary Table 4). Q443 mutant channels trended towards a reduced effect of DHA but still display a 99ms slowing in the decay rate. Meanwhile, mutation of E435 produced a 162ms slowing in the decay rate, which is comparable to the 168ms slowing that is produced in WT channels. Interestingly, the effect of DHA on the desensitization rate correlates well with the interaction frequency observed in our simulations: mutation of the residue with the most average contact numbers (R64) shows a total elimination of lipid modulation, while mutation of the residues with a combined second-most average contacts (Q60 and S434) showed a near total elimination of DHA regulation, and so forth (Fig. 4E, 5; Table 2, Supplementary Table 4). Taken together, these results suggest that while R63 serves as the primary anchoring site for the DHA headgroup, nearby residues such as Q59, Q441, and S432 form a network of secondary stabilizing interactions that help orient and retain DHA near the outer leaflet of the open-state ASIC3 channel.

### Identified DHA-ASIC3 interactions do not explain effects on pH-dependent channel activation:

**Figure 6.**
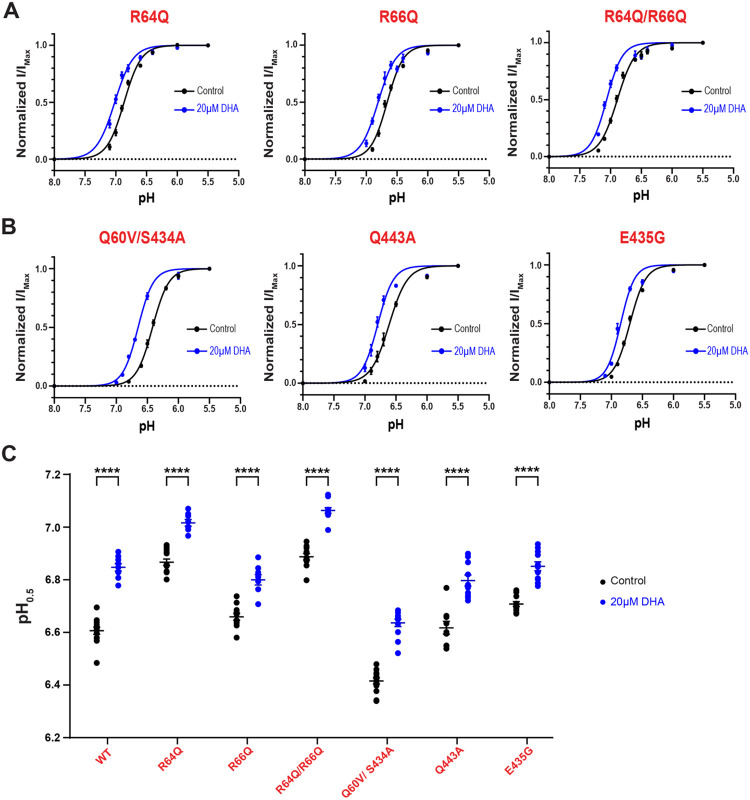
Mutations to TM residues are still impacted by the effect of DHA on pH-dependent channel activation. Fitted curves represent the pH dependence of channel activation of ASIC3 arginine mutants **(A)** and contact analysis mutants **(B)** ± 20μM DHA, fitted using a Hill-type equation (see Equation 1 in Methods). **(C)** Average activation pH_0.5_ values for ASIC3 mutant channels in response to 0 and 20μM DHA. ASIC3 WT data are replotted from Fig. 1 for comparison. All data given as mean ± SEM. C, unpaired t-test with Welch’s correction. ****, P < 0.0001 (see Methods and Table 6 for details).

Single acyl chain lipids that regulate ASIC3 exert multiple effects on the channel. In addition to slowing desensitization, we have shown that many PUFAs shift the pH dependence of activation to more basic pHs. In principle, DHA could potentiate both effects through lipid interactions within the same region that we have now identified near the outer leaflet of the TMs. To test this, we examined the effect that 20μM DHA has on the pH dependence of activation in our group of ASIC3 mutants. Like what we have shown for WT, we plotted pH activation curves using currents from ASIC3 mutants that were elicited by brief pulses of varying pH solutions in the absence and presence of DHA (Supplementary Figure 5) and determined the half activating pH by fitting the curves to the hill equation (Fig. 6A–B).

Based on our findings thus far, we hypothesized that mutant channels that had a robust effect on disrupting DHA-mediated slowing in channel desensitization should also disrupt DHA’s ability to shift the pH dependence of channel activation towards more basic pHs. However, surprisingly all tested mutations still display significant shifts in the average pH_0.5_ values following application of 20μM DHA (Fig. 6C, Supplementary Table 5). It’s possible that this gating effect requires a more complex set of interactions that if we could make multiple mutations, we would eventually eliminate this effect. We attempted to make a triple-point mutation to Q60, R64, and S434; however, this proved to be detrimental to channel expression and function (Table 3). It is also possible that this network of interacting residues prevents simple mutants from eliminating interactions between DHA and ASIC3, and that the lipid alters channel function through a change in the local environment around the channel and not through a ligand-like interaction. Altogether though, this data suggests that binding to this region of charged and polar residues along the outer leaflet of the TMs largely does not influence the ability of DHA to produce alkaline shifts in the pH dependence of channel activation.

### NAAAs and LPCs may utilize a different binding mechanism to modulate ASIC3:

We and others have previously shown that ASIC3 can be modulated by at least three classes of lipids: PUFAS, NAAAs, and LPCs. NAAAs, like AG and arachidonoyl serine (AS), have even more potent effects on slowing the rate of channel desensitization when compared to PUFAs while LPCs produce the largest effects out of any lipid tested on ASICs thus far^3,14^. All three classes of lipids produce a slowing in the desensitization rate and an alkaline shift in the pH dependence of activation. It remains unclear though if these lipids, despite their structural differences, produce their functional effects through similar interaction mechanisms.

**Figure 7.**
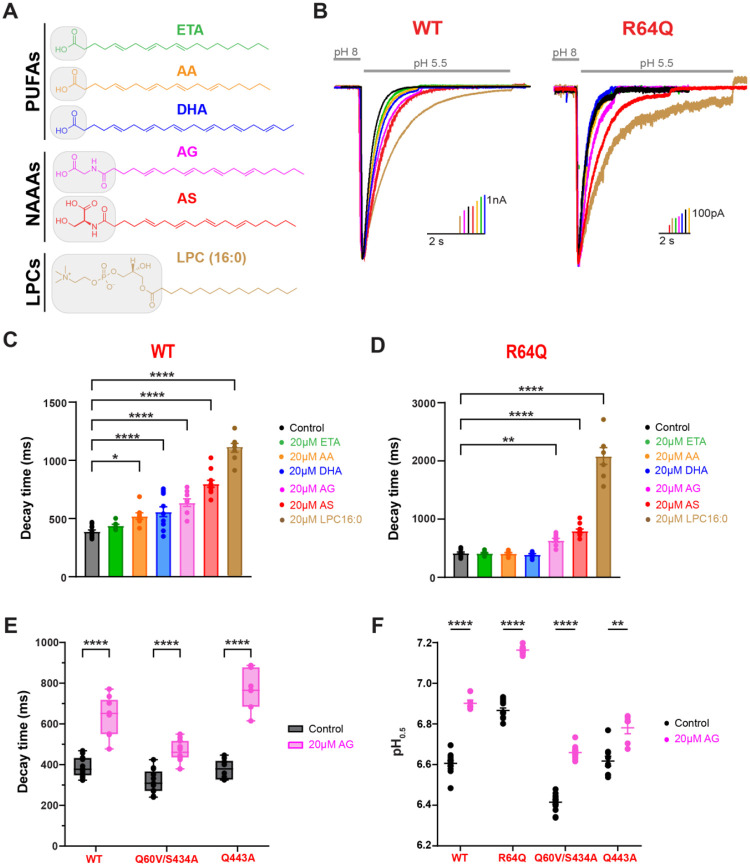
Mutations to TM residues disrupt PUFAs from slowing ASIC3 desensitization but not NAAAs of LPCs. **(A)** Chemical structures of PUFAs (ETA, AA, and DHA), NAAAs (AG and AS), and LPCs (LPC 16:0, which contain a 16-carbon chain with 0 cis double bonds) which were used in the following experiments. **(B)** Representative whole-cell recordings showing the slowing of the desensitization rate of ASIC3 WT (left) or R64Q (right) ± 20μM lipid. Currents were elicited by performing fast-switch perfusion changes between pH 8 and pH 5.5 solutions. **(C)** Bar graph showing the average time point at which ASIC3 WT currents decayed from their peak by 63% (1/e) at 0 and 20μM lipid. ASIC3 WT control and DHA data are replotted from Fig. 1 for comparison. **(D)** Bar graph showing the average time point at which ASIC3 R64Q currents decayed from their peak by 63% (1/e) at 0 and 20μM lipid. ASIC3 R64Q control and DHA data are replotted from Fig. 4 for comparison. **(E)** Box plot showing the average time point at which ASIC3 WT and contact analysis mutant currents decayed from their peak by 63% (1/e) ± 20μM AG application. ASIC3 WT control and AG data are replotted from Fig. 7C for comparison. **(F)** Average activation pH_0.5_ values for ASIC3 WT and mutant channels in response to 0 and 20μM AG. All data given as mean ± SEM. C-D, One-way ANOVA post hoc Dunnett’s test; E-F, unpaired t-test with Welch’s correction. *, P < 0.05; **, P < 0.01; ****, P < 0.0001 (see Methods and Table 7 for details).

To test this, we measured the effects of multiple lipids on R64Q mutant channels (Fig. 7A–B). We measured current upon solution switch from pH 8 to pH 5.5 and determined the time it takes the current to decay to 1/e of the peak value. Again, we preincubated our cells for at least 5 minutes with the indicated lipid prior to measuring currents. First, we tested three different PUFAs. AA and DHA are the most common PUFAs found within neuronal membranes, and both caused a significant slowing of ASIC3 WT desensitization (Fig. 7B–C, Supplementary Table 6). Surprisingly, eicosatrienoic acid (ETA) only showed a non-significant trend towards slowing desensitization rate despite previous work from our lab showing that it had the second largest effect on shifting ASIC3 proton sensitivity^3^. R64Q mutant channels showed no response to application of any of these three PUFAS at 20μM concentrations. We then tested two NAAAs (AG and AS), and both showed a robust slowing of channel desensitization in both WT and R64Q mutant channels (Fig.7B–D, Supplementary Table 6). Unexpectedly, R64Q channels showed an increased response to AS. Previous work identified two lysophosphatidylcholines, LPC(18:1) and LPC(16:0), as strong potentiators of ASIC3. Here we applied a mixture of LPCs which mainly contained the species LPC(16:0) to cells expressing either WT or R64Q mutant channels and found that both showed a dramatic slowing of channel desensitization. Like AS, LPC had an even larger effect on R64Q mutant channels compared to WT (Fig 7B–D, Supplementary Table 6). Because the head groups of NAAAs and LPCs tend to be larger and more electrically charged compared to that that seen in PUFAs (Fig. 7A), it’s possible that they utilize other interactions more within this region to stabilize themselves at the binding site. However, neutralizing other polar residues identified within this putative region did not impact AG-mediated effects on channel desensitization rate or pH dependence for channel activation either (Fig. 7E–F, Supplementary figure 5; Supplementary Tables 7,8). Altogether, our data suggests that the state dependent interaction with an arginine in TM1 is required for PUFAs to slow the desensitization rate of the channel but is not sufficient to explain how other potentiating lipids cause the same general gating change. Moreover, the shift in the pH dependence of activation does not require interaction with that arginine either.

## Discussion:

To date, there have been several studies showing the physiological consequences of lipid potentiation on ASIC currents^34–37^; by shifting the pH dependence for channel activation towards more physiological concentrations, neurons become more sensitized to firing action potentials following mild acidosis and in some cases in the absence of acidosis^35,37^. In addition to changes in proton sensitivity, there are also significant changes to channel desensitization rate. Here we examined the interaction of DHA with ASIC3 using MD simulations and then determined the functional relevance of those interactions using site-directed mutagenesis and patch-clamp electrophysiology. We found that an arginine residue that is only accessible to lipids in the open state, R63 in human ASIC3, was critical for both lipid interactions as well as the slowing of channel desensitization by DHA. When stably interacting with that arginine, the DHA head group makes a series of additional interactions that also help modulate changes in desensitization rate. In addition to this first ever description of a functional binding site for lipids on ASICs, our work made three surprising discoveries. First, other sites found in our simulations, while apparently important for interaction, did not have any impact on regulation of channel function by DHA. Second, none of the interactions we found, when mutated, impacted the ability of DHA to shift the pH dependence of channel activation. Finally, other classes of lipids known to potentiate ASICs do not require the same critical arginine as PUFAs, opening the possibility that there are other sites on the channel that sense changes in the lipid environment around the channel.

Our description of the binding interface here is largely consistent with growing evidence in the field. We previously suggested that this same general region in the outer portion of TM1 may be important for PUFA modulation of ASIC3 despite no direct evidence of binding^3^. This region included the arginine residue R63 (R64 in rat ASIC3) that we identify in this paper. Interestingly, R63 was not predicted to be involved in arachidonic acid binding *in-silico*, creating some discrepancies between computational and experimental data^29^. This previous computational study suggested that R65 and R68 were the critical binding site for arachidonic acid on ASIC3. Like these previous simulations, we found that positively charged arginine residues (R65 and R68 in human ASIC3) which face towards the membrane environment attract the negatively charged carboxyl head group of DHA molecules in both the resting and open state. However, the lack of arginines at these corresponding positions in rat ASIC3 had little effect on DHA regulation of channel activity. During our simulations, DHA molecules make transient but frequent interactions with R65 and R68. This results in an increase in the occupancy of DHA molecules near the channel, suggesting that the local lipid environment surrounding ASIC3 channels becomes enriched with DHA compared to the bulk. Unlike previous work, we found that when the channel transitions into an open state, DHA makes much longer-lived interactions with an arginine that is only exposed in the open state (R63). Helping to stabilize this interaction, the head group also makes contacts with nearby polar residues including Q59 and S432. We hypothesize, based on these results, that this state-dependent interaction is the mechanism by which DHA stabilizes the open state of ASIC3 by slowing the rate at which channels transition from a conducting state into a non-conducting state. While we cannot say for sure why our simulations found this stable interaction but previous ones did not, it may arise from the fact that our all-atoms MD simulations were simulated for 5μs, which provides a 10-fold longer sampling rate than what has been shown in previous work^29^. While the interaction with R63 is more stable, it also occurs less frequently, leaving open the possibility that this interaction is less likely to be detected during shorter simulations.

While we have identified a binding interface that can explain how PUFAs slow the rate of channel desensitization, it does not explain how PUFAs increase the proton sensitivity for channel activation. Mutations to this binding site were still able to produce shifts in the pH dependence in the basic direction following DHA application, suggesting that another binding mechanism may be involved. Although we did not identify another binding site within our simulations that were more stable than R63, it’s possible that PUFAs modulate different gating effects independently through multiple interaction sites. Work examining PUFA regulation of Kv7.1 channels has described a similar mechanism where differing gating effects are controlled through interactions with different sites on the channel^31^. Alternatively, this effect could be driven through a more complex set of interactions between PUFAs and ASIC3 at this binding interface, including those that are occurring along the tail group of the lipid. We previously found that PUFA potentiation of ASIC3 was largely driven by the electrostatic potential of the head group, however the properties of the tail group also had some impact on the potency of the lipid^3^. Within our simulations and others^29^, the PUFA carbon tails appear to position themselves along the TMDs. Protein-lipid interactions at these sites may serve to provide structural support for PUFAs to remain near the channel or to alter gating. We attempted several mutations at sites that our simulations showed interactions between the tail groups and the channel but mutating those residues resulted in nonfunctional channels.

It is also possible that the accumulation of DHA near the channel alters channel function through solvent-like effects on channel function. Solvent-like has been defined as non-ligand-like effects that occur through changes in local properties like bilayer fluidity or thickness. Our data does not suggest which solvent-like effect may be impacting the apparent sensitivity of the channel to protons. However, we have previously showed that the electronegativity of the lipid head group is critical for determining the magnitude of the effect of the lipid on ASIC3 function^3^. This increased electronegativity around the lipid-aqueous interface could have significant effects on the local pH near the channel^38,39^; negatively charged lipids surrounding the channel may accumulate more protons, which could result in a decrease in the local pH immediately surrounding the channel compared to the bulk pH. This would ultimately manifest as a shift in the pH dependence of channel activation in the basic direction. This also may explain the inability of our point mutations to eliminate the impact of DHA on the pH dependence of activation. Our mutation of R64Q in this study is not completely consistent with our previous result that did show a significant reduction in the impact DHA has on the proton sensitivity of the mutant channel. However, the data here shows a trend towards a reduction in the effect that DHA has on the pH dependence of activation and our previous work shows a significant but only partial reduction in the effect. We think that, likely, mutation of the more stable interaction sites on the channel could reduce the accumulation of these lipids around the channel and thus reduce the solvent-like effect on channel function.

While this study has focused primarily on PUFAs, there are other classes of single acyl chain lipids that we and others have shown to produce a slowing effect on the desensitization rate and an alkaline shift in the pH dependence of activation^3,35^. Given the impact that each of these classes of lipid has on channel function is qualitatively similar, it would be easy to hypothesize that these lipids share a common binding surface. However, we found that neutralizing the critical arginine, R64 in rat ASIC3, was only capable of disrupting PUFAs from slowing the rate of channel desensitization but was not sufficient to disrupt this effect produced by NAAAs or LPCs. Moreover, neutralizing other polar residues identified within this putative region did not impact AG-mediated effects on channel desensitization rate or pH dependence for channel activation. Surprisingly, the R64Q mutant channels showed an even larger effect on the slowing of desensitization rate following the application of AS and LPC. This could arise from an allosteric change in the protein lipid interaction due to some change in the structure or conformation of the channel. Differently, it could also suggest that these lipids may utilize the same binding surface. NAAAs have head group structures that contain the same carboxyl group as PUFAs but additional reactive groups. Arachidonoyl Serine, for instance, has an additional amino group and hydroxyl group that could increase its interaction surface which could prevent a single point mutation from being able to eliminate binding of these lipids to the channel. LPC is zwitterionic with both a phosphate and choline as part of its head group creating a much larger surface for potential interactions. In addition, AS and AG head groups are more electronegative than PUFA headgroups and LPC is thought to create a negative charge at the membrane/aqueous interface^38,39^. This would be consistent with our hypothesis that these accumulated lipids change the local pH around the channel as these more electronegative residues have larger impacts on channel function.

Increase of single acyl chain lipids like PUFAs and LPCs in the plasma membrane occurs in joint inflammation and leads to ASIC3-mediated pain^14^. Additionally, patients with rheumatic diseases that experience chronic pain and have in increased level of LPC in their synovial fluid are thought to feel pain through a mechanism involving ASIC3 potentiation via lipid^40^. Our description of a binding region for single acyl chain lipids on ASIC3 opens the door to searching for molecules that would inhibit binding of these lipids to this region of the channel and potentially reduce ASIC-mediated pain during joint inflammation. Our work here describes a relatively small binding site made up of a series of somewhat diffuse interaction sites for PUFAs on ASIC3 consistent with the idea that these lipids are likely acting through a combination of ligand-like and solvent-like effects. A competitor that blocked this region could eliminate all the potentiating effects of these lipids on ASIC3. However, more work is needed to understand of the other classes of potentiating lipids are acting through this same binding surface.

## Methods

### Materials and mutagenesis:

Docosahexaenoic acid (DHA; Cat. # 90310), N-Arachidonyl Glycine (AG; Cat. # 90051), Arachidonic Acid (AA; Cat. # 90010), 5(Z),8(Z),11(Z)-Eicosatrienoic Acid (ETA; Cat. # 90190), N-Arachidonoyl-L-Serine (AS; Cat. # 10005455) and Lysophosphatidylcholine (LPC; Cat. # 24331) were purchased from Cayman Chemical for use throughout all electrophysiology experiments. Rat ASIC3 plasmid was gifted by David Julius (University of California, San Francisco, San Francisco, CA) and subcloned into a pcDNA3.1 vector. The fluorescent tag mCerulean3 was attached to the C-terminus of the channel using a short proline rich linker to monitor its expression levels, which has been previously reported to have minimal effects on channel gating^41^. All mutant rat ASIC3 channels used in this study were made using site-directed mutagenesis either in house (KOD Hot Start Master Mix; Cat. #71842) or through Biozilla services. Successful mutagenesis was verified through whole plasmid sequencing services through Plasmidasaurus or sanger sequencing services through ACTG. Mutant plasmids were then maxiprepped using endotoxin-free methods either in house (Qiagen; Cat. # 12362) or through plasmid DNA purification services through Biozilla.

### Cell culture and Transfection:

CHO-K1 cells (ATCC) were cultured using Ham’s F12 medium supplemented with 10% FBS and were incubated at 37°C in 5% CO_2_. Cells grew to 70–80% confluency before being transfected with rat ASIC3 WT or mutant plasmid DNA (1.5–3μg). This was done either by electroporation with a Lonza 4D Nucleofector unit (Cat. # V4XC-1032) or by chemical transfection using TransIT transfection reagents (Cat. # MIR 2304) according to each manufacturer’s protocols. Following transfection, cells were plated on 12-mm glass coverslips coated in poly-L-lysine and incubated at 30°C in 5% CO_2_.

### Preparation and application of lipids:

All electrophysiological experiments which were in the presence of lipids were performed under identical conditions as control experiments except the resting pH 8 solutions contained the indicated concentration of lipid. All lipid stock solutions were made up in ethanol except in the case of LPC which was made up in a 2:1 ratio of chloroform: methanol (LPC) following the manufacturer’s recommendations. Ethanol or chloroform: methanol solvent in final solution was typically 0.01% and never exceeded 0.1%. Solution pH was measured prior to and after addition of PUFAs to solution to ensure no pH change occurred. For experiments that included lipid, cells were exposed to a holding pH 8 solution containing the indicated concentration of lipid for 5 minutes prior to eliciting pH activations unless stated otherwise to ensure any possible effects that were present under each condition would be saturated.

### Electrophysiological recordings:

As previously described^3,42^, all experiments were performed 16–30 h after transfection. To assess peak current magnitudes, whole-cell patch-clamp configuration was used. Borosilicate glass pipettes (Harvard Apparatus) were pulled to a resistance of 2–4MΩ (P-1000; Sutter Instrument) for whole-cell experiments. Glass pipettes were filled with an internal solution containing (in mM) 20 EGTA, 10 HEPES, 50 CsCl, 10 NaCl, and 60 CsF, pH 7.2. Extracellular solution contained (in mM) 110 NaCl, 5 KCl, 40 NMDG, 10 MES, 10 HEPES, 5 glucose, 10 Trizma base, 2 CaCl2, and 1 MgCl2, and pH was adjusted with HCl or NaOH as needed. An Axopatch 200B amplifier and pCLAMP 10.6 (Axon Instruments) were used to record whole-cell rASIC3 WT and mutant currents. All recordings were performed at a holding potential of −80 mV with a 5-kHz low-pass filter and sampling at 10 kHz. Solution changes were performed using a rapid perfusion system (SF-77B Fast-Step perfusion, Warner Instruments). The fluorescence of each recorded cell was visualized on an Olympus IX73 microscope with a CoolLED pE-4000 illumination system.

#### Desensitization rate:

To measure desensitization rates in the absence and presence of DHA and other PUFAs, cells were exposed to a holding pH of 8 for 8 s with or without lipid followed by a 2-s application of pH 5.5. For experiments on WT channels including AS and LPC applications, cells were exposed to a holding pH of 8 for 14 s followed by a 6-s application of pH 5.5. For experiments on R64Q channels including AS application, cells were exposed to the same conditions as WT channels while those including LPC were exposed to a holding pH of 8 for 20 s followed by 8-s applications of pH 5.5 to ensure the current decay was mostly saturated. Exposure to pH 8 solution between pH 5.5-activating pulses was also increased to ensure full recovery of the channels expressed on the cell membrane between each sweep. Rates were determined by taking the time point at which 1/e (63%) of the peak of pH 5.5 current had decayed. Each individual experiment was measured across multiple sweeps that were then averaged to obtain an average decay time for that experiment.

#### Shifts in pH-dependence of activation:

To determine the pH dependence of channel activation, a series of sweeps were measured from a holding pH of 8 for 8 s, followed by 2-s activation pulses at indicated pHs starting from a maximally activating pH of 5.5 down to a minimally activating pH which was dependent on construct and lipid condition. Each activation pH was measured for two sweeps which were averaged together and then were normalized to the responses elicited at pH 5.5.

#### Wash-on rate:

To measure the increase in current amplitude over time, cells in the absence of PUFAs were exposed to a holding pH of 8 for 4 seconds followed by a 1-s minimally activating pulse of pH 6.8. Cells were only exposed to lipid conditions at the initiation of each experiment, except in the case of experiments where internal application of lipid was achieved. In this case, cells were exposed to internal application of lipid for no more than 30 seconds prior to the start of each experiment to ensure that seals remained stable following break in. Data was normalized by comparing each of the responses within each experiment to the peak current within the first sweep.

### Data and statistical analysis:

PUFA structure schematics provided within the figures were created using MarvinSketch chemical editing software (ChemAxon). ClustalW multiple sequence alignment was performed using Jalview 2.11.5.0 software and manually edited in Adobe Illustrator (2025). Sequences were retrieved from Uniprot using the following Uniprot IDs: Q9UHC3, human ASIC3; O35240, rat ASIC3; Q62962, rat ASIC2; P55926, rat ASIC1; P78348, human ASIC1. Whole-cell patch clamp current recordings were analyzed using Clampfit 10.6 (Axon Instruments). All statistical data are plotted as mean ± SEM.

For pH dependence, reported pH_0.5_ values represent the mean values for each individual experiment as determined by fitting to a Hill-type equation in GraphPad:

(1) $$I=\frac{1}{{1+10}^{\left[(pH0.5-pHx)n\right]}}$$

where *n* is the Hill number and pH_0.5_ equals the half-maximal activating pH. Plotted fits were restrained to a maximum value of 1 and a minimum value of 0.

Statistical analyses were performed using GraphPad Prism 10 software. P values are reported in tables as calculated except for p-values < 0.0001, where they are reported as such. Figure legends indicate which statistical test was performed for each data set. For figures, statistical significance is indicated by the following scale: *, P < 0.05; **, P < 0.01; ***, P < 0.001; ****, P < 0.0001. All p-values reported account for multiple comparisons using appropriate tests. For all statistical testing requiring multiple comparisons within each construct, a One-way ANOVA was performed followed by post hoc testing. P values reported in Tables 1–4, and 7 represent Dunnett’s multiple comparisons test performed on all lipid data sets in the table compared with the control ASIC3 dataset. Unpaired student’s t tests were performed where indicated for statistical testing between two sets of data within each construct (e.g., Tables 5–6, S2–3). All t tests were two-tailed, and unpaired t tests assumed unequal variance.

### Homology Modeling and Molecular Docking:

Since no experimentally resolved structures for human ASIC3 are available in the Protein Data Bank (PDB), homology modeling was used to construct its structure in the open state. The chicken ASIC1 structure in the open state (PDB ID: 4NTY) and the human ASIC1 structure in the resting state (PDB ID: 7CFS) were employed as templates for modeling. The protein sequence of hASIC3 was retrieved from the UniProt database (UniProt ID: Q9UHC3) and submitted to the Swiss-Model web server (version 1.2). Swiss-Model generated 12 homology models for the open and resting state of hASIC3. Model selection was based on the Global Model Quality Estimate (GMQE) score^43^, which provides a quality measure for predicted structures. The model with the highest GMQE score was selected to represent both the open and resting states of hASIC3. This structure was then used for molecular docking and for building simulation systems for molecular dynamics (MD) studies.

Molecular docking was performed in the Schrödinger suite^44^. Using the Protein Preparation Wizard, the protein structure was pre-processed by assigning bond orders, adding hydrogens, adjusting protonation states at pH 7.4, creating disulfide bonds, and removing water molecules. Hydrogen-bond optimization and restrained minimization were subsequently performed. The 3D structure of docosahexaenoic acid (DHA) was obtained from PubChem. LigPrep^44^ was used to generate possible ligand protonation states between pH 6.0 and 8.0 and to assign chirality. R63 was selected as the receptor grid center based on our first simulation in this study and previous studies identifying R63 as a critical residue for PUFA potentiation in rat ASIC3^3,29,42^. For the ligand midpoint box, dimensions were set to 30 × 30 × 30 Å. Docking was carried out using the Glide XP (extra precision) mode in Maestro^44^. The docked complexes were analyzed in the Pose Viewer, and the best-scoring poses (based on G-scores) were selected for MD simulations. Because ASIC3 is a homotrimer with three equivalent lipid-binding sites, three DHA molecules were docked to the R63 site of the open-state ASIC3 model and subsequently subjected to MD simulations to investigate binding interactions.

### Molecular Dynamics Simulation System Settings:

All simulation systems were constructed using the CHARMM-GUI Membrane Builder^45,46^. Disulfide bonds were added between cysteine residue pairs (C92–C186, C164–C171, C282–C370, C315–C366, C319–C364, C328–C350, and C330–C342) to preserve the structural integrity and stability of the protein. Protonation states of ionizable residues were determined by pKa predictions from PROPKA3 at pH 7.4^47^, and residues were assigned to their default states accordingly. The protein was oriented along the bilayer normal (z-axis) using PPM 2.0 to ensure accurate alignment and helix tilt within the membrane^48^. The protein was then embedded into the lipid bilayer (Table 1), and each system was solvated in a 0.15 M NaCl aqueous solution using CHARMM-GUI protocols^45^. Trajectories 1 and 2, corresponding to the resting- and open-state hASIC3 models, were simulated in a POPC bilayer with a POPC:DHA ratio of 10:1, where DHA molecules were randomly distributed across both leaflets. Although this enrichment exceeds physiological concentrations, it enabled systematic sampling of DHA–protein contacts and reproducible quantification of lipid occupancy and interaction lifetimes without compromising bilayer stability. The simulation boxes for Trajectories 1 and 2 measured 103 × 103 × 142 Å^3^ and 105 × 105 × 138 Å^3^, containing approximately 200,000 and 182,000 atoms, respectively. For Trajectory 3, a 3-DHA–docked open-state hASIC3 structure was embedded in a pure POPC bilayer under identical solvation and ionic conditions, with a box size of 105 × 105 × 135 Å^3^ and a total of ~175,000 atoms, allowing direct comparison of DHA binding events between spontaneous and pre-bound systems.

### All-atom MD Simulation protocol:

All-atom MD simulations were performed using either NAMD 2.14^49^ or the specialized computational platform Anton2^50^. The CHARMM36m force field was used for proteins, lipids, and ions within the study^51–55^. The explicit representation of water molecules was achieved through the implementation of the TIP3P model^56^. The simulations were executed under NPT (constant number of particle N, pressure P, and temperature T) conditions at a temperature of 310 K and a pressure of 1 atm, utilizing periodic boundary conditions. Electrostatic interactions were handled using the particle mesh Ewald method with a 12 Å cutoff for real-space interactions^57^. Hydrogen atom bond distances were constrained through the application of the SHAKE algorithm^58^. Hydrogen mass repartitioning (HMR) allows us to re-distribute the mass of heavy atoms to the bonded hydrogens^59^. Hence a time step of 4 fs was selected for the simulations ran on NAMD. Prior to the production runs, minimization and equilibration were performed in NAMD 2.14 with harmonic positional restraints applied in two sequential stages. In the first stage, restraints were imposed on all protein heavy atoms, allowing solvent and lipids to relax around a fixed protein framework. In the second stage, the restraints were reduced to include only protein Cα atoms and ligand heavy atoms, permitting sidechain and lipid rearrangements while maintaining the overall backbone geometry. Each simulation had 5,000 steps of energy minimization followed by two-stage NPT equilibration for a combined duration of 2 ns. The equilibrated systems were simulated at microseconds timescale. In MD simulations conducted with Desmond on Anton2, a Berendsen coupling scheme was implemented to sustain a consistent pressure of 1.0 atm. The calculation of long-range electrostatic interactions was facilitated by the k-space Gaussian split Ewald method^60^, utilizing a 64 × 64 × 64 grid. Time-step for simulations run on Anton2 is 2.5 fs. MD trajectories were then visualized and analyzed using VMD^61^, in-house Tcl, and Python scripts.

#### Occupancy map of DHA:

To quantify and visualize the preferred spatial distribution of DHA molecules surrounding the hASIC3 channel, time-averaged occupancy maps were generated using the VolMap plug-in in VMD 1.9.4^61^. Occupancy maps were computed for Trajectory 1 (resting state) and Trajectory 2 (open state) to capture state-dependent differences in DHA localization. The analysis focused on DHA carboxylate carbon atoms, which mediates electrostatic and hydrogen-bonding interactions with the protein surface. A 1 Å grid resolution was used, and the resulting volumetric density was normalized to yield an occupancy map, reflecting the frequency with which DHA head groups occupied each spatial grid element during the simulation. The maps were visualized at 0.3, corresponding to regions where DHA head-group atoms were present for at least 30 % of the total trajectory, revealing persistent DHA-binding zones near the arginine-rich region of hASIC3.

#### Binding lifetime analysis:

A binding event was defined as a continuous time interval during which the carboxylate carbon of a DHA molecule remained within 5 Å of the sidechain of residues R63, R65, or R68. For comparison, other nearby residues within the TM domain were analyzed using the same criterion. Brief interruptions of fewer than 10 consecutive frames were merged into the same event to avoid artificial fragmentation. For each residue, we calculated the following metrics: total number of events (N), mean event duration (ns), and longest individual binding event (ns). A summary of binding lifetime statistics is presented in Table 2.

#### Contact analysis:

Contact analysis was carried out using the inter-sel contacts function within the Timeline plug-in of VMD 1.9.4^61^. This analysis identified all residues of hASIC3 that formed close interactions with carboxylate carbon of DHA molecules throughout trajectory 3. A cutoff distance of 4 Å between heavy atoms was applied to define a contact, ensuring that both transient and stable interactions were captured. The contact number was calculated as the total number of atom pairs between DHA and each protein residue at a given time frame, providing a quantitative measure of lipid–protein interactions across the trajectory. This approach enabled the identification of residues most frequently involved in DHA binding. This analysis identified all residues of hASIC3 that formed close interactions with carboxylate carbon of DHA molecules throughout trajectory 3. A cutoff distance of 4 Å between heavy atoms was applied to define a contact, ensuring that both transient and stable interactions were captured. The contact number was calculated as the total number of atom pairs between DHA and each protein residue at a given time frame, providing a quantitative measure of lipid–protein interactions across the trajectory. This approach enabled the identification of residues most frequently involved in DHA binding.

## Supplementary Material

1

## Figures and Tables

**Table 1:** Summary of MD simulations performed throughout study.

MD trajectories ID	PUFA in system	Lipid Composition	State	Length of Simulation (μs)
1	DHA partitioned in a lipid layer	POPC + DHA (10:1)	resting	5
2	DHA partitioned in a lipid layer	POPC + DHA (10:1)	open	5
3	3 DHA-docked	POPC	open	0.5

**Table 2:** Summary of DHA binding lifetimes with key ASIC3 residues in the resting and open states. The total number of binding events, mean binding duration, and longest individual binding event were calculated for residues located near the outer leaflet of the TM domains during 5-μs MD simulations in both resting and open states.

hASIC3 Residue	State	Total events	Mean Duration (ns)	Longest event (ns)
*Y58*	Resting	23	18.6	61
	Open	12	18	42
*Q59*	Resting	8	42	107
	Open	14	26	89
*R63*	Resting	--	--	--
	Open	21	495.3	3246
*R65*	Resting	130	162.2	1196
	Open	168	141.7	1232
*R68*	Resting	142	124.5	2891
	Open	191	113	1437
*S432*	Resting	9	49.1	130
	Open	50	24.4	175
*Q441*	Resting	--	--	--
	Open	78	27.2	532
*E433*	Resting	--	--	--
	Open	--	--	--

**Table 3:** Non-functional/ poorly expressing mutants.

rASIC3 Construct	Observable Currents?	Above 100pA?
*Q60V*	0/8 cells	0/8 cells
*Q60V/R64Q/S434A*	9/20 cells	0/20 cells
*L446A/F447C*	0/7 cells	0/7 cells
