# Supplementary material for: PUFA modulation of ASIC3 involves both specific and lipid solvent-like interactions
Source: bioRxiv. 2026 Jan 2:2026.01.02.697424. Preprint. [Version 1] doi: 10.64898/2026.01.02.697424 (PMC12776306; doi:10.64898/2026.01.02.697424)
Supplement: 1 [file NIHPP2026.01.02.697424V1-supplement-1.pdf]

bioRxiv preprint doi: https://doi.org/10.64898/2026.01.02.697424; this version posted January 2, 2026. The copyright holder for this preprint (which was not certified by peer review) is the author/funder, who has granted bioRxiv a license to display the preprint in perpetuity. It is made available under aCC-BY 4.0 International license.

Supplementary Information

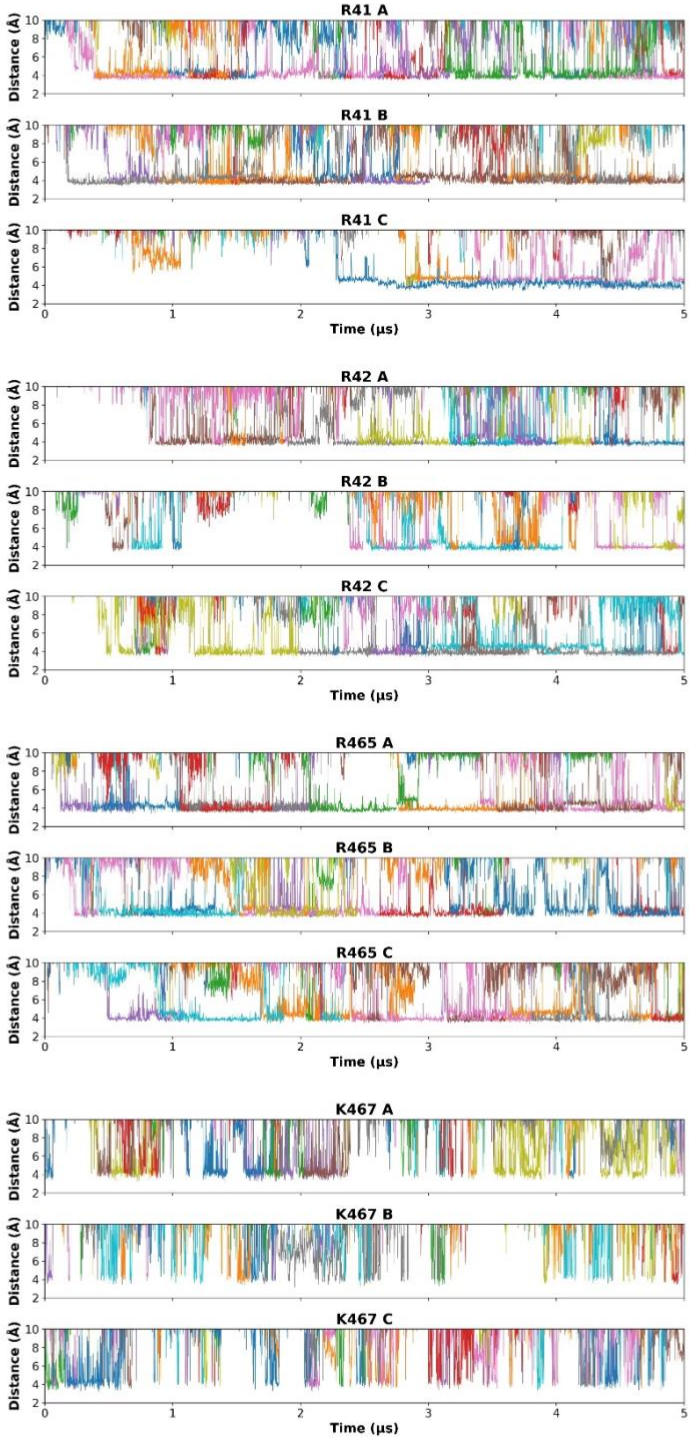

Supplementary Figure 1: The spontaneous DHA binding to residues near inner leaflet in resting-state (Traj. 1): Distance plots showing the proximity between the carboxylate carbon of DHA and the terminal carbon of the side chains of residues near inner leaflet include R41, R42, R465, and K467. Different colored traces correspond to distinct DHA molecules. Only DHA molecules that approached within 5 Å of a given residue in at least one frame were included in the analysis.

bioRxiv preprint doi: https://doi.org/10.64898/2026.01.02.697424; this version posted January 2, 2026. The copyright holder for this preprint (which was not certified by peer review) is the author/funder, who has granted bioRxiv a license to display the preprint in perpetuity. It is made available under aCC-BY 4.0 International license.

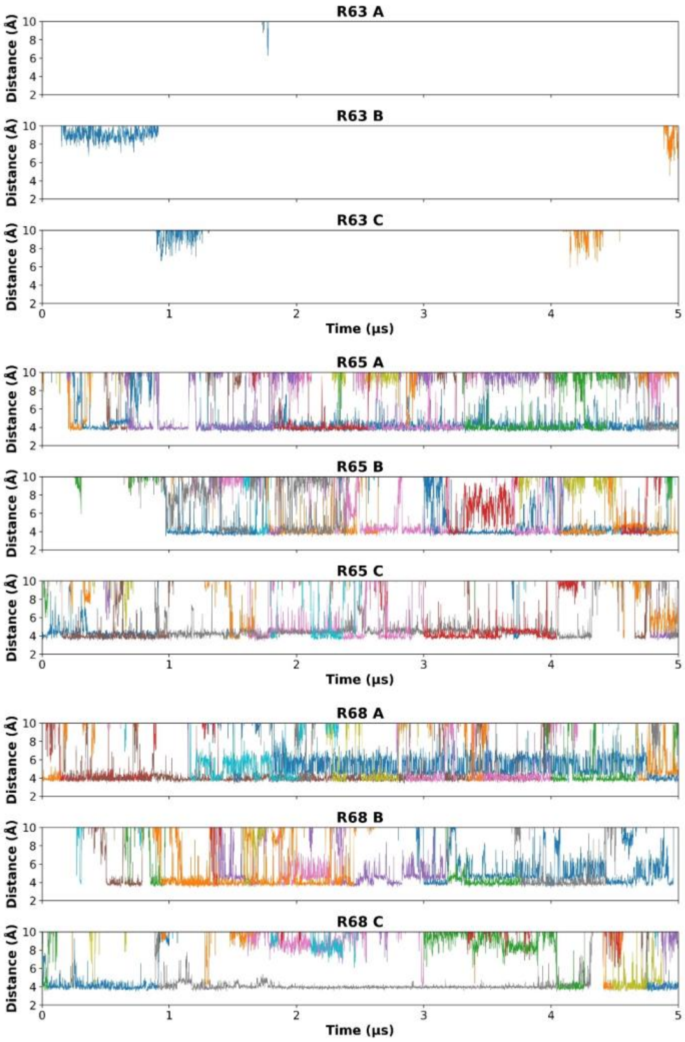

Supplementary Figure 2: The spontaneous DHA binding to residues near outer leaflet in resting-state (Traj. 1): Distance plots showing the proximity between the carboxylate carbon of DHA and the terminal carbon of the side chains of arginine residues near outer leaflet (R63, R65 and R68) Different colored traces correspond to distinct DHA molecules. Only DHA molecules that approached within 5 Å of a given residue in at least one frame were included in the analysis.

bioRxiv preprint doi: https://doi.org/10.64898/2026.01.02.697424; this version posted January 2, 2026. The copyright holder for this preprint (which was not certified by peer review) is the author/funder, who has granted bioRxiv a license to display the preprint in perpetuity. It is made available under aCC-BY 4.0 International license.

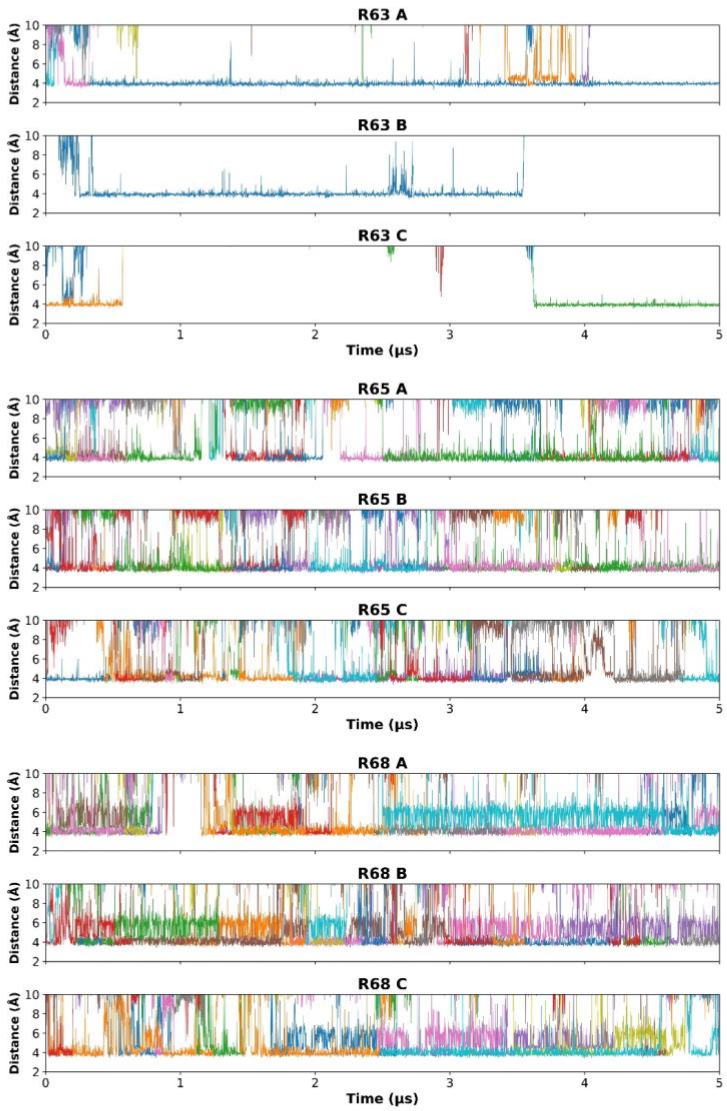

Supplementary Figure 3: The spontaneous DHA binding in open state (Traj. 2): Distance plots showing the proximity between the carboxylate carbon of DHA and the terminal carbon of the side chains of upper-leaflet residues R63, R65, and R68. Different colored traces correspond to distinct DHA molecules. Only DHA molecules that approached within 5 Å of a given residue in at least one frame were included in the analysis.

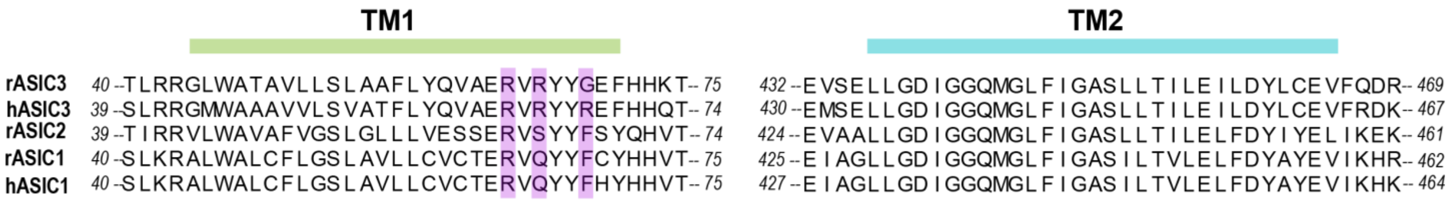

Supplementary Figure 4: Sequence alignment for the segments TM1 and TM2 for rat ASIC3, human ASIC3, rat ASIC2, rat ASIC1, and human ASIC1. Highlighted in purple are the residues in TM1 that align with the critical arginine residues identified in rat and human ASIC3 across all ASIC genes.

bioRxiv preprint doi: https://doi.org/10.64898/2026.01.02.697424; this version posted January 2, 2026. The copyright holder for this preprint (which was not certified by peer review) is the author/funder, who has granted bioRxiv a license to display the preprint in perpetuity. It is made available under aCC-BY 4.0 International license.

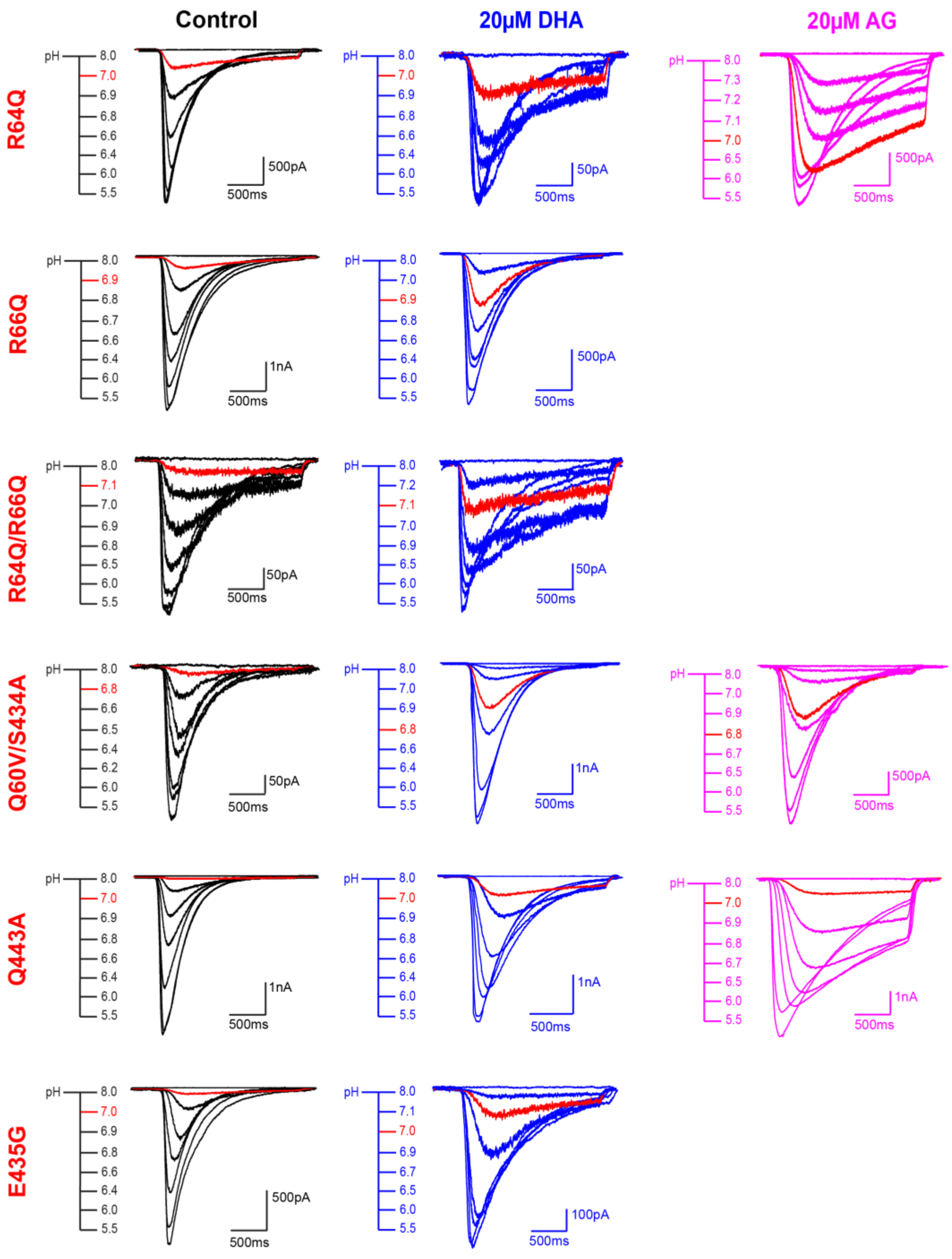

Supplementary Figure 5: Representative traces of rat ASIC3 WT and mutants showing pH-dependent channel activation ± 20µM DHA or AG application. Traces highlighted in red are to indicate the response to a specific pH across all conditions within each construct. For lipid conditions, cells were pre-incubated for at least 5 minutes prior to each experiment.

bioRxiv preprint doi: https://doi.org/10.64898/2026.01.02.697424; this version posted January 2, 2026. The copyright holder for this preprint (which was not certified by peer review) is the author/funder, who has granted bioRxiv a license to display the preprint in perpetuity. It is made available under aCC-BY 4.0 International license.

Supplementary Table 1: Decay rates (1/e) and activation $pH_{0.5}$ values for rASIC3 WT ± 10 and 20µM DHA. *n* denotes the number of individual cells. Statistical analysis was performed using a One-way ANOVA with Dunnett's post-hoc multiple comparisons test. Mean differences and adjusted P values were obtained by comparing control vs. lipid conditions.

rASIC3 Construct	Condition	1/e (ms)	SEM	*n*	Mean diff. (Control vs. Lipid)	Adjusted P value	$pH_{0.5}$	SEM	Mean diff. (Control vs. Lipid)	Adjusted P value
WT	Control	389.90	11.51	16	--	--	6.606	0.0137	--	--
	10µM DHA	485.72	33.96	9	-95.82	0.0560	6.779	0.0094	-0.1727	<0.0001
	20µM DHA	558.14	42.15	12	-168.2	0.0002	6.847	0.0136	-0.2410	<0.0001

bioRxiv preprint doi: https://doi.org/10.64898/2026.01.02.697424; this version posted January 2, 2026. The copyright holder for this preprint (which was not certified by peer review) is the author/funder, who has granted bioRxiv a license to display the preprint in perpetuity. It is made available under aCC-BY 4.0 International license.

rASIC3 Construct	Condition	1/e (ms)	SEM	*n*	Mean diff. (Control vs. Lipid)	Adjusted P value	$pH_{0.5}$	SEM	Mean diff. (Control vs. Lipid)	Adjusted P value
WT	Control	389.90	11.51	16	--	--	6.606	0.0137	--	--
	20μM DHA (internal; 5 min)	366.8	12.82	10	23.10	>0.9999	6.605	0.0181	0.001	0.9952
	20μM DHA (internal; 10 min)	402.1	12.63	10	-12.22	>0.9999	6.627	0.0192	-0.0214	0.8815
	20μM DHA (external)	558.14	42.15	12	-168.2	<0.0001	6.847	0.0136	-0.2410	<0.0001

Supplementary Table 2: Decay rates (1/e) and activation $pH_{0.5}$ values for rASIC3 ± 20μM DHA applied internally vs. externally. *n* denotes the number of individual cells. Statistical analysis was performed using a One-way ANOVA with Dunnett's post-hoc multiple comparisons test. Mean differences and adjusted P values were obtained by comparing control vs. lipid conditions. WT control and 20μM DHA external data from Table 1 was used for comparison.

bioRxiv preprint doi: https://doi.org/10.64898/2026.01.02.697424; this version posted January 2, 2026. The copyright holder for this preprint (which was not certified by peer review) is the author/funder, who has granted bioRxiv a license to display the preprint in perpetuity. It is made available under aCC-BY 4.0 International license.

rASIC3 Construct	Condition	1/e (ms)	SEM	*n*	Mean diff. (Control vs. Lipid)	Adjusted P value
R64Q	Control	418.93	16.22	16	--	--
	10µM DHA	389.79	13.38	13	18.76	0.2805
	20µM DHA	413.63	13.13	8	-5.076	0.9659
R66Q	Control	435.44	17.76	11	--	--
	10µM DHA	503.97	19.73	13	-68.52	0.0274
	20µM DHA	519.16	18.70	10	-83.72	0.0107
R64Q/R66Q	Control	497.67	34.97	11	--	--
	10µM DHA	488.90	18.97	12	8.770	0.9571
	20µM DHA	505.37	20.66	10	-7.701	0.9696

Supplementary Table 3: Decay rates (1/e) for rASIC3 arginine mutants ± 10 and 20µM DHA application. *n* denotes the number of individual cells. Statistical analysis was performed within each construct using a One-way ANOVA with Dunnett's post-hoc multiple comparisons test. Mean differences and adjusted P values within each rASIC3 construct were obtained by comparing control vs. lipid conditions.

bioRxiv preprint doi: https://doi.org/10.64898/2026.01.02.697424; this version posted January 2, 2026. The copyright holder for this preprint (which was not certified by peer review) is the author/funder, who has granted bioRxiv a license to display the preprint in perpetuity. It is made available under aCC-BY 4.0 International license.

rASIC3 Construct	Condition	1/e (ms)	SEM	*n*	Mean diff. (Control vs. Lipid)	Adjusted P value
WT	Control	389.90	11.51	16	-168.2	0.0084
	20μM DHA	558.14	42.15	12		
Q60V/S434A	Control	317.96	16.10	13	-31.39	0.1214
	20μM DHA	349.35	10.90	13		
Q443A	Control	317.96	16.10	13	-99.32	0.0039
	20μM DHA	349.35	10.90	13		
E435G	Control	438.40	8.50	13	-162.5	<0.0001
	20μM DHA	600.94	25.86	10		

Supplementary Table 4: Decay rates (1/e) for rASIC3 WT and contact analysis mutants ± 20μM DHA application. *n* denotes the number of individual cells. Statistical analysis was performed within each construct using unpaired t-tests with Welch's correction. Mean differences and adjusted P values within each rASIC3 construct were obtained by comparing control vs. 20μM DHA. WT control and 20μM DHA data from Table 1 was used for comparison.

bioRxiv preprint doi: https://doi.org/10.64898/2026.01.02.697424; this version posted January 2, 2026. The copyright holder for this preprint (which was not certified by peer review) is the author/funder, who has granted bioRxiv a license to display the preprint in perpetuity. It is made available under aCC-BY 4.0 International license.

rASIC3 Construct	Condition	$pH_{0.5}$	SEM	*n*	Mean diff. (Control vs. Lipid)	Adjusted P value
R64Q	Control	6.866	0.0121	13	-0.1499	<0.0001
	20μM DHA	7.016	0.0125	8		
R66Q	Control	6.659	0.013	11	-0.1401	<0.0001
	20μM DHA	6.799	0.019	8		
R64Q/R66Q	Control	6.887	0.012	11	-0.1758	0.0003
	20μM DHA	7.063	0.0107	11		
Q60V/S434A	Control	6.415	0.0118	13	-0.2209	<0.0001
	20μM DHA	6.636	0.0141	13		
Q443A	Control	6.617	0.0246	9	-0.1794	0.0003
	20μM DHA	6.797	0.0212	10		
E435G	Control	6.708	0.0091	12	-0.1433	<0.0001
	20μM DHA	6.851	0.0170	11		

Supplementary Table 5: Activation $pH_{0.5}$ values for rASIC3 mutants ± 20μM DHA application. *n* denotes the number of individual cells. Statistical analysis was performed within each construct using unpaired t-tests with Welch's correction. Mean differences and adjusted P values within each rASIC3 construct were obtained by comparing control vs. 20μM DHA.

bioRxiv preprint doi: https://doi.org/10.64898/2026.01.02.697424; this version posted January 2, 2026. The copyright holder for this preprint (which was not certified by peer review) is the author/funder, who has granted bioRxiv a license to display the preprint in perpetuity. It is made available under aCC-BY 4.0 International license.

rASIC3 Construct	Condition	1/e (ms)	SEM	*n*	Mean diff. (Control vs. Lipid)	Adjusted P value
WT	Control	389.90	11.51	16	--	--
	20µM ETA	440.07	10.56	8	-50.17	0.7065
	20µM AA	521.39	27.78	8	-131.5	0.0103
	20µM DHA	558.14	42.15	12	-168.2	<0.0001
	20µM AG	636.87	31.90	9	-247.0	<0.0001
	20µM AS	799.96	30.016	11	-410.1	<0.0001
	20µM LPC	1107.44	37.63	8	-717.5	<0.0001
R64Q	Control	418.93	16.21	16	--	--
	20µM ETA	392.92	16.30	9	26.01	0.9967
	20µM AA	410.67	16.33	8	8.253	>0.9999
	20µM DHA	413.63	13.13	8	5.297	>0.9999
	20µM AG	613.59	46.36	8	-194.7	0.0018
	20µM AS	1078.05	58.06	11	-659.1	<0.0001
	20µM LPC	2084.75	143.55	7	-1666	<0.0001

Supplementary Table 6: Decay rates (1/e) for rASIC3 WT and R64Q mutant channels ± 20µM lipid applications. *n* denotes the number of individual cells. Statistical analysis was performed within each construct using a One-way ANOVA with Dunnett's post-hoc multiple comparisons test. Mean differences and adjusted P values within each rASIC3 construct were obtained by comparing control vs. lipid conditions. WT and R64QWT control and 20µM DHA data from Tables 1 and 4 respectively were used for comparison.

bioRxiv preprint doi: https://doi.org/10.64898/2026.01.02.697424; this version posted January 2, 2026. The copyright holder for this preprint (which was not certified by peer review) is the author/funder, who has granted bioRxiv a license to display the preprint in perpetuity. It is made available under aCC-BY 4.0 International license.

rASIC3 Construct	Condition	1/e (ms)	SEM	*n*	Mean diff. (Control vs. Lipid)	Adjusted P value
WT	Control	389.90	11.51	16	-247.0	<0.0001
	20μM AG	636.87	31.90	9		
Q60V/S434A	Control	318.0	16.10	13	-151.0	<0.0001
	20μM AG	469.0	12.94	14		
Q443A	Control	377.7	15.07	10	-450.1	<0.0001
	20μM AG	768.1	36.92	7		

Supplementary Table 7: Decay rates (1/e) for ASIC3 WT and mutants ± 20μM AG application. *n* denotes the number of individual cells. Statistical analysis was performed within each construct using unpaired t-tests with Welch's correction. Mean differences and adjusted P values within each ASIC3 construct were obtained by comparing control vs. 20μM AG. WT data from Table 7 was used for comparison.

bioRxiv preprint doi: https://doi.org/10.64898/2026.01.02.697424; this version posted January 2, 2026. The copyright holder for this preprint (which was not certified by peer review) is the author/funder, who has granted bioRxiv a license to display the preprint in perpetuity. It is made available under aCC-BY 4.0 International license.

rASIC3 Construct	Condition	$pH_{0.5}$	SEM	*n*	Mean diff. (Control vs. Lipid)	Adjusted P value
WT	Control	6.606	0.014	13	-0.2562	<0.0001
	20µM AG	6.862	0.027	5		
R64Q	Control	6.866	0.012	13	-0.2625	<0.0001
	20µM AG	7.129	0.028	8		
Q60V/S434A	Control	6.415	0.012	13	-0.2113	<0.0001
	20µM AG	6.627	0.010	13		
Q443A	Control	6.617	0.025	9	-0.1507	0.0049
	20µM AG	6.768	0.026	6		

Supplementary Table 8: Activation $pH_{0.5}$ values for ASIC3 WT and mutants ± 20µM AG application. *n* denotes the number of individual cells. Statistical analysis was performed within each construct using unpaired t-tests with Welch's correction. Mean differences and adjusted P values within each ASIC3 construct were obtained by comparing control vs. 20µM AG.
